# Editorial Note: Knowledge and practice of clients on preventive measures of COVID-19 pandemic among governmental health facilities in South Wollo, Ethiopia: A facility-based cross-sectional study

**DOI:** 10.1371/journal.pone.0326898

**Published:** 2025-06-23

**Authors:** The PLOS One Editors

This article [1] was identified as one of a series of articles for which the *PLOS One* Editors have concerns about authorship and adherence to the journal’s first and second publication criteria. We regret that the issues were not addressed prior to the article’s publication.
